# Robust Data Augmentation Generative Adversarial Network for Object Detection

**DOI:** 10.3390/s23010157

**Published:** 2022-12-23

**Authors:** Hyungtak Lee, Seongju Kang, Kwangsue Chung

**Affiliations:** 1 School of Computer and Information Engineering, Kwangwoon University, Seoul 01897, Republic of Korea; 2 Department of Electronics and Communications Engineering, Kwangwoon University, Seoul 01897, Republic of Korea

**Keywords:** generative adversarial network, data augmentation, object detection, image-to-image translation, disentangled representation learning

## Abstract

Generative adversarial network (GAN)-based data augmentation is used to enhance the performance of object detection models. It comprises two stages: training the GAN generator to learn the distribution of a small target dataset, and sampling data from the trained generator to enhance model performance. In this paper, we propose a pipelined model, called robust data augmentation GAN (RDAGAN), that aims to augment small datasets used for object detection. First, clean images and a small datasets containing images from various domains are input into the RDAGAN, which then generates images that are similar to those in the input dataset. Thereafter, it divides the image generation task into two networks: an object generation network and image translation network. The object generation network generates images of the objects located within the bounding boxes of the input dataset and the image translation network merges these images with clean images. A quantitative experiment confirmed that the generated images improve the YOLOv5 model’s fire detection performance. A comparative evaluation showed that RDAGAN can maintain the background information of input images and localize the object generation location. Moreover, ablation studies demonstrated that all components and objects included in the RDAGAN play pivotal roles.

## 1. Introduction

Neural network-based object detection models outperform traditional ones and have become a milestone in object detection techniques. However, the prodigious performance of neural network-based models is derived from millions of parameters and a tremendous amount of training data, which allow sufficient training of models. MS COCO and Pascal VOC are well-known datasets in general object detection tasks [1,2]. These datasets contain numerous images of ordinary objects and accurate annotations, which alleviates the concerns of researchers regarding the datasets they use and allows them to focus on their research. However, creating high-quality datasets is a labor-intensive, time-consuming, and expensive task. Therefore, datasets for infrequently occurring incidents are insufficient. This lack of datasets triggers small-dataset and class imbalance problems, which limits the model performance.

Image data augmentation methods have been proposed increase the dataset size at a lower cost by using images from existing datasets. One approach to image data augmentation is basic image manipulation, which involves simple operations such as cropping or flipping images. Although this approach has a low computational cost and can increase the size of the dataset, it can cause overfitting problems if the size of the dataset is insufficient [3]. Another method is image data augmentation using deep learning, and the most frequently used ones are based on generative adversarial networks (GANs). After training the GAN model with the image dataset to be augmented, these approaches augment the dataset by sampling the images from the trained model. Some studies have proposed image data augmentation methods based on GAN [4], and they obtain better object detection performance than those trained with the existing dataset.

Although any object can be targeted through GAN-based image data augmentation, most studies have been conducted in the medical field [4]. One reason is that it is difficult to obtain a large amount of data for medical images because of the characteristics of the medical domain, such as privacy and disease rarity. Images of fire experience a similar data shortage problem. In the case of a fire, it is difficult to generate data owing to safety issues, and existing datasets comprise an insufficient number of images. Moreover, unlike general objects, fire is difficult to create because a flame is an unstructured object without clear edges. Therefore, a smooth transition between the fire and the background is important. Additionally, because a fire results from burning an object, many real-world fire images appear blended with background objects. Therefore, if there is an object at the location targeted for flame insertion, the approach to create a fire image using the cut-and-paste method is not suitable because the object and fire should appear naturally harmonized.

**Figure 1:**
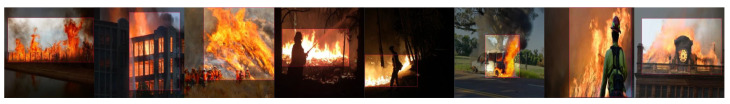
Examples of the images in the dataset used in this study. Most images exhibit strong occlusions.

This study targets labeled data generation by augmenting an object detection dataset with a small amount of general data. Structured objects have sufficient data and are easy to label, but unstructured objects do not. To alleviate these problems, we focus on improving the detection performance by data augmentation when a small dataset of an unstructured object is given. We select fire as the representative of unstructured objects. In this study, we used fire images showing various fire situations, as shown in Figure 1. These images contain occlusions, and the scale variances of flames are significant. To solve these problems, we propose a novel GAN-based model for data augmentation, called the robust data augmentation GAN (RDAGAN). RDAGAN converts clean fire-free images into fire images using a dataset containing a small number of fire images with strong occlusions. Because the generated images are used for training object detection models, RDAGAN must insert a flame into the target area that will be used as the bounding box for the generated images. By making the best use of the given dataset, it generates realistic fire images that can enhance the performance of object detection models. The contributions of this study can be summarized as follows.

1. We propose RDAGAN to solve the small-dataset problem in object detection tasks. The RDAGAN includes two networks: an object generation network and an image translation network. The object generation network reduces the burden on the image translation network by generating a flame patch, which acts as a guideline for the image translation network. The image translation network translates the entire image into a fire scene by blending the generated flames and clean images.
2. We propose the concept of information loss that binds two networks by maximizing the mutual information of the outputs of the two networks to retain the information of the generated flame patch in the image translation network.
3. Background loss is proposed to improve the performance of RDAGAN. The background loss compares the difference between an input image and the image generated through the image translation network, and makes them as similar as possible. Consequently, the generated images have sharp edges and diverse color distributions.
4. A quantitative experiment demonstrates that the dataset augmented using RDAGAN can achieve better flame detection performance than baseline models. Moreover, through comparative experiments and ablation studies, we show that RDAGAN can generate labeled data for fire images.

## 2. Related Work

### 2.1. Disentangled Representation Learning and InfoGAN

Disentangled representation learning is an unsupervised learning technique. Its goal is to find a disentangled representation that affects only one aspect of the data, while leaving others untouched [5].

To find a disentangled representations, InfoGAN [6] was proposed, which is a variation of GAN that finds interpretable disentangled representations instead of unknown noise. InfoGAN allows the model to learn a disentangled representation by employing constraints during representation learning. Moreover, it divides the input into incompressible noise and latent code, and maximizes the mutual information between the latent code and generator distribution. That is, the latent code information is retained during the generation process.

### 2.2. Image-to-Image Translation

The Image-to-Image (I2I) translation technique maps images of one domain to another. Although this task may seem similar to style transfer, they have a key difference. Style transfer aims to translate images such that they have the style of one target image while maintaining the contents of the image. In contrast, I2I translation aims to create a map between groups of images [7].

Pix2Pix [8] was the first supervised I2I conditional GAN-based model used for learning mappings between two paired image groups. However, because Pix2Pix has an objective function based on the L1 loss between translated and real images, unpaired datasets cannot be used for training. Unsupervised I2I translation models have been proposed to solve this problem. Cycle-consistent adversarial network (CycleGAN) [9] is one of the best-known unsupervised I2I translation models. It contains two pairs of generators and discriminators. Each generator and discriminator pair learns to map an image onto the opposite domain. Additionally, cycle-consistency loss have been proposed, which is defined using the L1 distance between the original image and that recovered from the image translated into another domain. Cycle-consistency loss can alleviate the problem caused by the absence of a paired dataset [10]. Contrastive learning for unpaired image-to-image translation (CUT) [11] is an unsupervised I2I translation model based on contrastive learning. Its goal is to ensure that a patch of translated image contains the content of the input image. CUT achieves this goal by maximizing mutual information through contrastive loss. Contrastive loss maximizes the similarity of patches for the same location in both the input and output images and minimizes patch similarity at different locations.

### 2.3. GAN-Based Image Data Augmentation

An image data augmentation method based on GAN is widely used in many fields such as medical imaging or remote sensing. These fields are hard to obtain sufficient data in to train neural networks because they require a large amount of training data. When the number of data points is small, it is easy for models to be overfitted or fall into the class imbalance problem. The GAN-based image data augmentation methods can relieve these problems by generating new samples from a data distribution. Frid-Adar et al. proposed a method to generate liver lesion images using GANs [12]. In the study, even though they utilized only 182 liver lesion computed tomography images to train the GAN model, the performance of a convolutional neural network-based model was improved for liver lesion classification.

Furthermore, because the I2I translation model translates an image of one domain into that of another, some studies have generated labeled datasets for object detection and image segmentation using I2I translation. In this case, the target domain of translation becomes the dataset to be augmented, and the image that is the subject of the translation becomes the source image. Lv et al. proposed a GAN-based model augmenting remote sensing images for the image segmentation task [13]. They proposed deeply supervised GAN (D-sGAN) that automatically generates remote sensing images and their labels. The D-sGAN accepts random noise and target segmentation maps, and synthesizes remote sensing images corresponding to the input segmentation map. The generated images from D-sGAN increased the remote sensing interpretation model accuracy by 9%. Pedestrian-Synthesis-GAN (PS-GAN) [14] was proposed to reduce the cost of pedestrian image annotations. The PS-GAN uses an image with inserted noise. The object is placed in the noisy area, a pedestrian image is inserted into a noise box, and the generated image is evaluated using one discriminator for the entire image and another for the generated pedestrian patch. The dataset augmented using this model has been shown to improve the detection performance of a region-based convolutional neural network [15].

### 2.4. Fire-Image Generation for Image Data Augmentation

Few studies have been conducted on creating fire images for specific tasks. Some of them were studied to improve performance of fire classification. Campose and Silva proposed a CycleGAN-based model to translates non-fire aerial images into aerial ones with fire [16]. This model translates non-fire aerial images into aerial ones with fire. The model works based on the cut-and-paste algorithm to control the fire generation area. Park et al. proposed a CycleGAN-based model to relieve a class imbalance problem for wildfire detection [17]. This model translates non-fire images into wildfire ones. Because image classification tasks do not require annotations for fire regions, there is no need to control flame generation. Thus, these studies used the barely modified CycleGAN architectures that are only able to translate clean images to fire images.

Other studies have been conducted to improve image segmentation performance. Yang et al. proposed a model for creating a flame image to improve the flame segmentation performance in a warehouse [18]. A limitation of their study is that the boundary between the square area and the background of a generated image can be clearly distinguished because the model performs image translation only on the square area around the inserted flame. Qin et al. proposed a model for creating realistic fire images, including the effects of flames [19]. Their model uses a cut-and-paste algorithm to paste the flame onto the image and then creates natural fire images that include light-source effects, such as halo by image translation. In this study, more natural fire images were created by solving the problems encountered in previous studies. Both studies had limitations in that, rather than modeling general fire images, they only considered indoor images, and the images had little clutter and occlusion between the flame and background objects.

## 3. Methods and Materials

In this section, we introduce the proposed RDAGAN model. The goal was to build a model that maps clean images in a clean image domain ($i_{c}\in C$) to target images in the target image domain ($i_{t}\in T$). The proposed model was trained using an object detection dataset containing few images, and most images had occlusions.

The proposed model employs the divide-and-conquer approach, where the model is divided into two networks: an object generation network and an image translation network. The model not only endeavors to insert realistic object in the image ($i_{c}$) but also transforms the entire image to appear such as those in the target domain (T). These goals are hard to be achieved using a single GAN model because the training becomes unstable.

### 3.1. Object Generation Network

The object generation network creates an image of the target object to be inserted into $i_{c}$. The image generated from the object generation network is used as an input for the image translation network. The image mitigates the training instability in the image translation network owing to the goals of object creation and image translation. The network adopts the InfoGAN [6] architecture to obtain a disentangled representation of the target object. The disentangled representation obtained from the object generation network is used in the image translation network to build a loss function. We trained the network with object images $R(i_{t})$ that were cropped and resized from image $i_{c}$ using the crop and resize module *R*.

**Figure 2:**
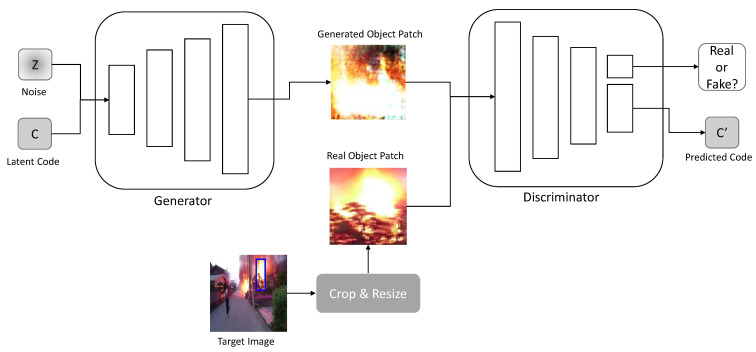
Architecture of the object generation network.

As shown in Figure 2, generator $G_{obj}$ accepts the incompressible noise *z* and latent code *c* as inputs, which are sampled from a normal distribution. The discriminator $D_{obj}$ not only validates the input images but predicts the input latent code $c^{\prime }$.

#### Objectives

Because the object generation network uses the InfoGAN architecture, the model objective $L^{obj}$ comprises two losses: adversarial loss $L_{GAN}^{obj}$ and information loss $L_{Info}^{obj}$.

The adversarial loss $L_{GAN}^{obj}$ [20] is used to make the generated patch $G_{obj}\left(z,c\right)$ similar to that of the target domain images $R(i_{t})$ as follows:

(1) $$L_{GAN}^{obj}=E_{R(i_{t})∼T}\log D_{obj}\left(R\left(i_{t}\right)\right)+E_{R(i_{c})∼C}\log\left(1-D_{obj}\left(G_{obj}\left(z,c\right)\right)\right)$$

Information loss $L_{Info}^{obj}$ [6] measures the mutual information between latent code *c* and generated image $G(R\left(i_{c}\right))$. It is calculated using the mean squared error of input latent code *c* and predicted code $c^{\prime }$ from the discriminator $D_{obj}$, as follows:

(2) $$L_{Info}^{obj}=E_{c∼N\left(0,1\right),c^{\prime }∼D_{obj}\left(G_{obj}\left(z,c\right)\right)}(||c-c^{\prime }{\left|\right|}_{2})$$

The full objective $L^{obj}$ is the sum of previous losses:

(3) $$L^{obj}=L_{GAN}^{obj}+\lambda L_{Info}^{obj}$$

where λ represents the strength of the information loss. The model was trained by minimizing the full objective.

### 3.2. Image Translation Network

The image translation network merges the clean images $i_{c}\in C$ and object patch $G_{obj}\left(z,c\right)$ generated from the object patch network, while making image $i_{c}$ similar to the target image $i_{t}\in T$. However, it is challenging to perform these complicated tasks simultaneously using the vanilla GAN model [20] and a single adversarial loss. Hence, the proposed model includes a local discriminator [21] and additional loss functions to reduce the burdens of complicated tasks.

#### 3.2.1. Generator

**Figure 3:**
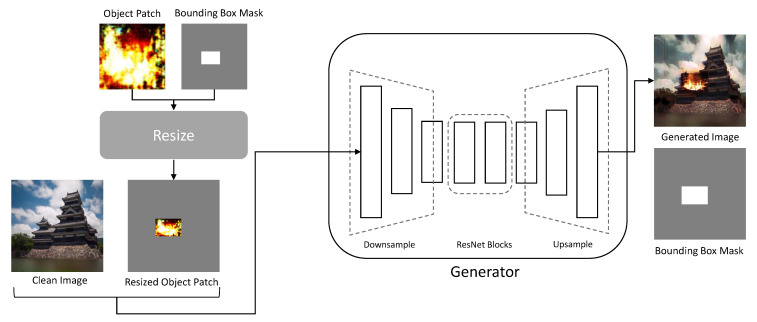
Structure of the the image translation network generator.

As shown in Figure 3, the the image translation network generator $G_{tr}$ has an encoder–decoder architecture comprising residual network (ResNet) [22] blocks in the middle, similar to the generator used in CycleGAN [9]. However, unlike [23], the generator has flexibility in the shape variance of the generated image because all features are downsampled and upsampled.

To create the image, the generator requires a bounding box mask $m_{b}$, which indicates the location of flame insertion. As shown in Equation (4), the position where the value of the mask is 0 indicates the background, and that where the value is 1 indicates the flame. There are no particular algorithms used to determine the bounding box region. Each point of the bounding box area is randomly sampled from the discrete uniform random distribution within the height and width of the images.

(4) $$m_{b}=\left\{\begin{array}{ccc} 0 & \mathrm{for} & \mathrm{background}\\ 1 & \mathrm{for} & \mathrm{flame} \end{array}\right.$$

The resized object patch $i_{p}:=Resize\left(G_{obj}\left(z,c\right)\right)$ is obtained by resizing the object patch, wherein the patch is positioned in the area where the value of the bounding box mask is one. The resized object patch is concatenated with a clean image and used as the generator input. The generator creates the generated image $G_{tr}\left(i_{p},i_{c}\right)$ by naturally blending the six-channel combined images and translating them such that they are similar to the target domain image $i_{t}\in T$

#### 3.2.2. Discriminator

**Figure 4:**
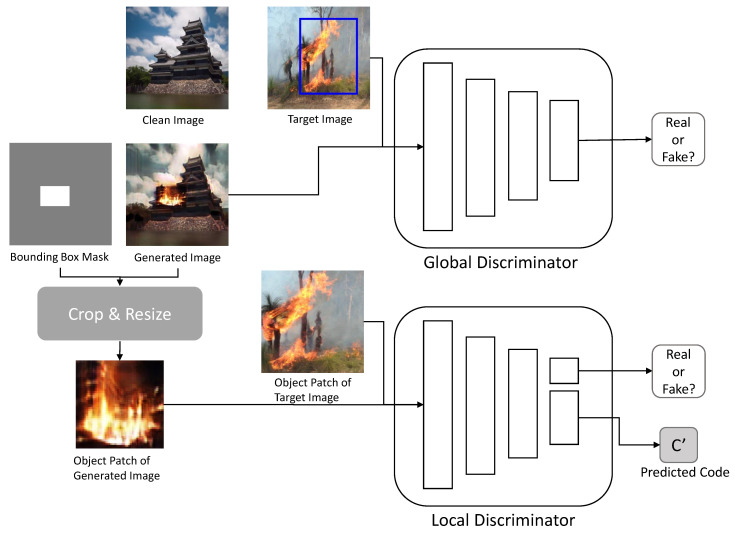
Structure of the discriminators in the image translation network.

As shown in Figure 4, the image translation network comprises two discriminators: global $D_{tr}^{global}$ and local $D_{tr}^{local}$. These discriminators perform the image translation network tasks of image translation and natural blending.

The global discriminator $D_{tr}^{global}$ evaluates the images $G_{tr}\left(i_{p},i_{c}\right)$ generated by the generator. Its structure is based on the PatchGAN [8] discriminator that evaluates patches of the image rather than the whole one. It evaluates whether the image is similar to the image of the target domain image T. This evaluation result constitutes an adversarial loss.

The local discriminator $D_{tr}^{local}$ determines whether the object patch $R(G_{tr}\left(i_{p},i_{c}\right))$ is realistic, and whether the object patch $R(G_{tr}\left(i_{p},i_{c}\right))$ can be obtained through the cropping and resizing operation *R* using the mask of the generated image $G_{tr}\left(i_{p},i_{c}\right)$. The structure of the local discriminator is similar to the structure of the global discriminator. However, like the InfoGAN [6] discriminator, it contains an additional auxiliary layer that produces the predicted code $c^{\prime }$ from feature maps of the image. The authenticity evaluation result of the local discriminator is contained in the adversarial loss, and the predicted code is used to construct an information loss.

#### 3.2.3. Adversarial Loss

We used adversarial loss $L_{GAN}^{tr}$ [20] to allow the generator to learn the mapping from C to T. The objective is expressed as follows:

(5) $$\begin{array}{c} \begin{array}{cc} L_{GAN}^{tr} & =E_{i_{t}∼P_{T}}\log D_{tr}^{global}\left(i_{t}\right)\\ \; & +E_{i_{p}∼G_{gen}\left(z,c\right),i_{c}∼P_{C}}\log D_{tr}^{local}\left(G_{tr}\left(i_{p},i_{c}\right)\right)\\ \; & +E_{i_{t}∼P_{T}}\log\left(1-D_{tr}^{global}\left(R\left(i_{t}\right)\right)\right)\\ \; & +E_{i_{p}∼G_{gen}\left(z,c\right),i_{c}∼P_{C}}\log(1-D_{tr}^{local}\left(R\left(G_{tr}\left(i_{p},i_{c}\right)\right)\right) \end{array} \end{array}$$

where $G_{tr}$ tries to generate images similar to those obtained from the target domain T and target objects appear as real objects, whereas the global discriminator $D_{tr}^{global}$ aims to distinguish the generated image $G_{tr}\left(i_{p},i_{t}\right)$ from the images obtained from T. The local discriminator $D_{tr}^{local}$ endeavors to differentiate the generated object $R(G_{tr}\left(i_{p},i_{t}\right))$ from the object obtained from T.

#### 3.2.4. Information Loss

The goal of the image translation network cannot be achieved using adversarial loss alone because the target image $i_{t}$ contains both target objects and occlusions. Therefore, the local discriminator simultaneously learns not only the shape and texture of the object itself, but also occlusions caused by other objects. This hinders the generator from using and blending the object patch $i_{p}$ with the clean image $i_{c}$ and creates artifacts in the generated images. Additionally, it causes the generator to fall into mode collapse. To solve this problem, we introduce information loss to constrain the input object patch $G_{obj}\left(z,c\right)$ and the cropped object of the generated image $R(G_{tr}\left(i_{p},i_{c}\right))$ to have similar characteristics, which allows the generator to blend the object patch $i_{p}$ with the clean image $i_{c}$.

However, it is difficult to create two images with similar characteristics by directly using the input object patch $i_{p}$ and the generated image object patch $R(G_{tr}\left(i_{p},i_{c}\right))$. Therefore, we achieved this by maximizing the mutual information between the two. The mutual information is denoted as $I(i_{p};R\left(G_{tr}\left(i_{p},i_{c}\right)\right))$, where I(X;Y) is the mutual information between random variables *X* and *Y*. The mutual information is defined as H(X)−H(X|Y), where H(X) and H(X|Y) are the marginal and conditional entropies, respectively.

Maximizing $I(i_{p};R\left(G_{tr}\left(i_{p},i_{c}\right)\right))$ is also problematic, because $i_{p}$ and $R(G_{tr}\left(i_{p},i_{c}\right))$ have the same dimensionality. Maximizing $I(i_{p};R\left(G_{tr}\left(i_{p},i_{c}\right)\right))$ means making the two images as identical as possible, and it can be achieved by replacing the generated image patch $R(G_{tr}\left(i_{p},i_{c}\right))$ with $i_{p}$. Thus, we attempted to maximize $I(c;R\left(G_{tr}\left(i_{p},i_{c}\right)\right))$ instead of $I(i_{p};R\left(G_{tr}\left(i_{p},i_{c}\right)\right))$. This is because the object generation network $G_{gen}$ is trained to maximize the mutual information between *c* and $i_{p}$. In [6], it was demonstrated that maximizing the mutual information $I(c;R\left(G_{tr}\left(i_{p},i_{c}\right)\right))$ is the same as minimizing the difference between the latent code *c* and the predicted code $c^{\prime }$ from the local discriminator $D_{tr}^{local}$.

Therefore, we formulated the information loss $L_{Info}^{tr}$ as the difference between latent code *c* of $G_{gen}$ and predicted code $c^{\prime }$ of $D_{tr}^{local}\left(i_{p},i_{c}\right)$ using the mean squared error, and the objective is defined as follows:

(6) $$\begin{array}{c} \begin{array}{cc} L_{Info}^{tr} & =E_{c∼N\left(0,1\right),i_{p}∼G_{gen}\left(z,c\right),i_{c}∼P_{C}}(||c-D_{tr}^{local}\left(G_{tr}\left(i_{p},i_{c}\right)\right){\left|\right|}_{2})\\ \; & =E_{c∼N\left(0,1\right),c^{\prime }∼D_{tr}^{local}\left(G_{gen}\left(i_{p},i_{c}\right)\right)}(||c-c^{\prime }{\left|\right|}_{2}) \end{array} \end{array}$$

#### 3.2.5. Background Loss

The background loss was used to find the difference between the input image $i_{c}$ and the generated image $G_{tr}\left(i_{p},i_{c}\right)$, except for the bounding box mask area $m_{b}$. Owing to the nature of the generator $G_{tr}$ with an encoder-decoder structure, the image is first compressed into a low-dimensional representation and then recovered. This has the advantage in that the structure of the generated image is relatively free; however, there is a trade-off in that the fidelity of the image is lowered. Therefore, the edge components of the image are blurred, the tint of the image is significantly changed, and the color variance in the generated image is reduced.

To eliminate the reconstruction problem of the generator, background loss was introduced. Background loss is the pixel-wise L1 distance between the input clean image $i_{c}$ and the generated image $G_{tr}\left(i_{c},i_{p}\right)$, except for the mask $m_{b}$ area. This is because the flame merges in the region indicated by the mask. To exclude the flame region, we obtain the inverted mask $1-m_{b}$ and multiply it by the generated image $G_{tr}\left(i_{c},i_{p}\right)$ and the clean image $i_{c}$. The background loss strongly guides to the generator $G_{tr}$, stabilizes training, and allows $G_{tr}$ to produce sharp images [8]. The objective function $L_{BG}^{tr}$ is expressed as follows:

(7) $$L_{BG}^{tr}=E_{i_{c}∼C,m_{b}∼P_{m_{b}},i_{p}∼G_{gen}\left(z,c\right)}\left(|\right|\left(i_{c}*\left(1-m_{b}\right)\right)-\left(G_{tr}\left(i_{c},i_{p}\right)*\left(1-m_{b}\right)\right){\left|\right|}_{1})$$

#### 3.2.6. Full Objective

Finally, the full objective of the image translation network is formulated as follows:

(8) $$L^{tr}=L_{GAN}^{tr}+\lambda_{1}L_{BG}^{tr}+\lambda_{2}L_{Info}^{tr}$$

where $\lambda_{1}$ and $\lambda_{2}$ are the strengths of the background and information losses, respectively.

### 3.3. Overall Architecture

**Figure 5:**
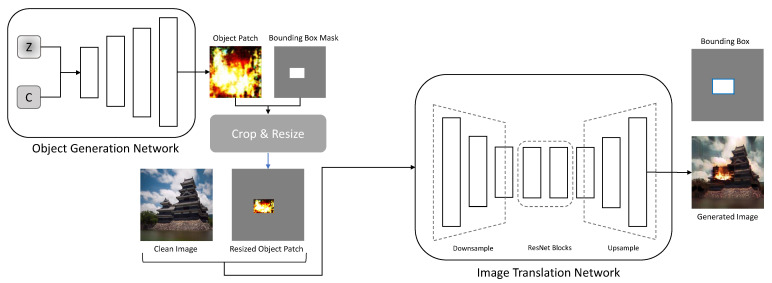
Overall architecture of RDAGAN.

The overall architecture of RDAGAN is shown in Figure 5. For RDAGAN data generation, the generators of the image generation and translation networks are used. $G_{obj}$ receives incompressible noise *z* and latent code *c* and creates an object patch $G_{obj}\left(z,c\right)$.

The RDAGAN samples the bounding box mask $m_{b}$ from the uniform distribution and uses it to create a resized object patch $i_{p}$. The resized object patch is passed to $G_{tr}$ with the clean image $i_{c}$, which is used as the background to create the generated image $G_{tr}\left(i_{p},i_{c}\right)∼i_{t}\in T$. After performing fire-image generation to generate images for the object detection dataset, mask $m_{b}$ is converted into a bounding box.

## 4. Experiments

We conducted qualitative and quantitative evaluations to demonstrate the image generation performance of RDAGAN and verify whether it can boost objective detection performance.

First, we designed a quantitative evaluation to prove that RDAGAN can generate labeled data that are sufficient to improve the detection performance of a deep learning model. We then performed a qualitative evaluation to confirm the image-generation ability of the image translation network. The qualitative evaluation comprised of a comparative evaluation and ablation studies. In the comparative evaluation, the abilities of the image translation model and baseline models were compared. In ablation studies, RDAGAN and its ablations were compared.

### 4.1. Implementation Details

For all the experiments, the object generation network included 112-dimensional noise and 16-dimensional latent code, and the size of the generated object patch was 128×128 pixels. The generator of the image translation network comprised two downsample layers, 11 ResNet blocks, and two upsample layers. The image translation network uses 256×256 pixel images for the generator and global discriminator and 64×64 pixel images as cropped object images for the local discriminator.

To evaluate the proposed model, we conducted experiments by using two datasets: FiSmo and Google Landmarks v2 dataset [24,25]. FiSmo dataset is a fire dataset that contains images of fire situations and annotations for object detection and segmentation task. In experiments, we used images and bounding boxes of FiSmo dataset as the source of fire images. Google Landmarks v2 dataset is a large-scale dataset comprising about 5 million landmark images. The Google Landmarks v2 dataset was used as a non-fire background image for generating fire images in our model.

In the quantitative experiment, the YOLOv5 [26] model with 86.7 million parameters was used to evaluate the object detection performance. Two datasets were constructed to train the models: one dataset comprising 800 images sampled from the FiSmo dataset, and the other comprising images augmented from the first dataset. The second dataset was composed of 800 FiSmo images and 3000 images sampled from RDAGAN. To test the YOLOv5 model, a dataset with 200 images sampled from the FiSmo dataset was used.

In the qualitative evaluation, the FiSmo dataset was used as the target image dataset to train all models. The Google Landmarks v2 dataset was used as the clean image dataset. For training the RDAGAN, we used 1500 samples that were randomly selected from the datasets. For generating images through RDAGAN, images sampled from the Google Landmarks v2 dataset were used as input. None of the images in the datasets used in the experiments overlapped with the others.

The baseline models used in the comparative experiment were the CycleGAN [9] and CUT [11], which are widely used unsupervised I2I translation models. To ensure a fair comparison, we provided object patches and clean images to the network during training. These patches reduce the burden of object generation. For CycleGAN, the generator network was provided with an additional object mask, which mapped the target domain T to a clean image domain C. This allowed the network to locate the target object easily.

### 4.2. Quantitative Evaluation

For the quantitative evaluation, the YOLOv5 model was trained using the FiSmo dataset and that augmented using RDAGAN. The augmented dataset was inflated with images sampled using RDAGAN, which was trained with the same datasets as those used in the comparative experiment. We evaluated the performance of the trained models to confirm whether the generated images and bounding boxes could improve the detection performance.

#### 4.2.1. Evaluation Metrics

To evaluate the proposed model, we focused on the accuracy of the YOLOv5 model. We adopted four metrics to measure the accuracy of the YOLOv5 model: precision, recall, F1 score, and average precision (AP). Object detection includes two subtasks: bounding box-regression and object classification. We evaluated the classification performance by measuring the precision and recall. The bounding box regression capacity can be scaled using the AP.

Precision is the percentage of true positives (tp) among the total number of true and false positives (fp). Recall is the percentage of (tp) among the total number of (tp) and false negatives (fn). These metrics are calculated as follows:

(9) $$precision=\frac{tp}{tp+fp}$$

(10) $$recall=\frac{tp}{tp+tn}$$

Precision and recall vary with the confidence threshold of the detector. In this evaluation, we set the threshold as the value at which the F1 score was maximized.

There is a trade-off relationship between precision and recall. That is, in most cases, if precision increases, recall is suppressed. To evaluate the classification results, the F1 score can be used as a holistic evaluation metric of accuracy instead of precision and recall. It can be derived by calculating the harmonic mean of precision and recall as follows:

(11) $$F1=2*\frac{precision*recall}{precision+recall}$$

Owing to the trade-off relationship between precision and recall, we instead used the F1 score to quantify the results.

Average precision (AP) is a widely used precision metric for evaluating object detection models. The AP is obtained by computing the area of the precision–recall curve obtained by varying the model confidence [27]. It can be considered with the overlap threshold, intersection over union (IOU), which is defined as the fraction of the intersection of the overlapped area between the ground truth bounding box $b_{gt}$ and the predicted bounding box $b_{p}$ over the union of the areas [2] as follows:

(12) $$\mathrm{IOU}=\frac{area(b_{p}\cap b_{gt})}{area(b_{p}\cup b_{gt})}$$

Using the IOU threshold, predictions wherein IOUs are less than the threshold are considered false positives [27]. We obtained AP by applying two IOU threshold settings. In the first setting, the IOU threshold was set to 0.5, and in the other, it varied from 0.5–0.95 with a step size of 0.5. We denote these IOUs as AP@0.5 and AP@0.5:0.95, respectively.

#### 4.2.2. Comparative Experiment

We compared the images and the object patches generated by RDAGAN and baseline models, CycleGAN and CUT. We evaluated the translation of the entire image, and the localization and quality of the generated flame.

### 4.3. Ablation Studies

#### 4.3.1. Image Generation

We compared the images generated by RDAGAN with those generated by its ablations. The ablations included four models with various parts eliminated: one without the background loss, one without the object patches and information loss, one without the local discriminator and information loss, and one without the object patches and local discriminator.

#### 4.3.2. Object Generation

The importance of information loss, background loss, and the local discriminator was evaluated by comparing the objects generated by RDAGAN and its ablations.

## 5. Results and Discussion

### 5.1. Quantitative Evaluation Results

Table 1 lists the performance of the trained YOLOv5 model. The dataset augmented with the data generated through RDAGAN shows an improvement in AP@0.5 from 0.5082 to 0.5493 and in AP@0.5:0.95 from 0.2917 to 0.3182, wherein the IOU threshold ranged from 0.5–0.95.

Although the recall of the model trained with the augmented dataset was slightly decreased by 2.6%, the precision showed a substantial improvement from 0.5497 to 0.6922, which was an improvement of 14.2%. Moreover, the F1 score of the model trained with augmented data increased from 0.5465 to 0.5921. Thus, RDAGAN can augment data and increase the performance of object detection models without requiring additional target datasets or images.

### 5.2. Comparative Experiment Result

**Figure 6:**
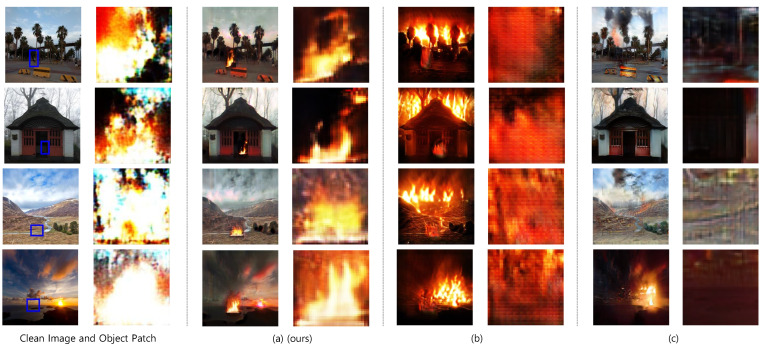
Images and object patches generated through RDAGAN (**a**), CycleGAN (**b**), and CUT (**c**). The target locations for flame insertion are indicated by squares.

Figure 6 shows the images and the object patches generated by the RDAGAN and baseline models. Figure 6a–c show the images and object patches generated using RDAGAN, CycleGAN, and CUT, respectively. We evaluated the translation of the entire image, and the localization and quality of the generated flame.

Regarding the translation of the entire image, RDAGAN showed a slight change in the image tint. However, it is evident that the overall characteristics of the background were maintained. In contrast, CycleGAN changed the entire image significantly. The area with the generated flame turned red and the background changed to a halo and became dark. Although CUT did not change the background of most images, it failed to generate flames in them. Regarding flame localization, RDAGAN generated a flame exactly within the given area, but CycleGAN generated flames in different locations and CUT either generated flames in a different locations or did not generate one at all. Moreover, CUT struggled to blend the flames; hence, only one sample in Figure 6c has a flame.

In conclusion, RDAGAN created flames exactly at the target locations while maintaining background characteristics. However, although CycleGAN generated flames in all images, the background was degraded and localization was completely ignored. Although some samples from CUT displayed flame and maintained the background characteristics to some extent, it obtained inadequate results for flame generation and localization.

### 5.3. Ablation Results

#### 5.3.1. Comparison of Image Generation Performance

**Figure 7:**
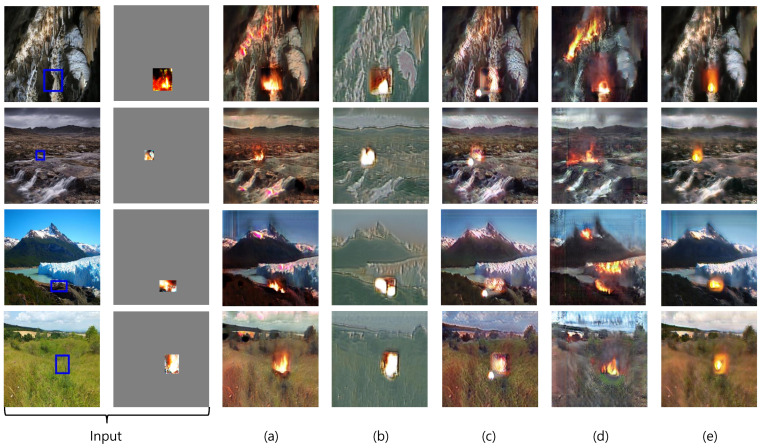
Generated images from RDAGAN and its ablations, (**a**) RDAGAN, (**b**) RDAGAN without $L_{BG}^{tr}$, (**c**) RDAGAN without $i_{p}$ and $L_{Info}^{tr}$, (**d**) RDAGAN without $D_{tr}^{local}$ and $L_{Info}^{tr}$, and (**e**) RDAGAN without $i_{p}$, $D_{tr}^{local}$, and $L_{Info}^{tr}$.

Figure 7 shows the image generation results of RDAGAN and its ablations. Figure 7a shows images generated by the RDAGAN, Figure 7b shows images generated by the model without $L_{BG}^{tr}$, Figure 7c shows images generated by the model without $i_{p}$ and $L_{Info}^{tr}$, Figure 7d shows images generated by the model without $D_{tr}^{local}$ and $L_{Info}^{tr}$, and Figure 7e shows images generated by the model without $i_{p}$, $D_{tr}^{local}$, and $L_{Info}^{tr}$. First, we evaluated the overall images and flame quality.

We compared the differences between the overall images generated by RDAGAN and its ablations. In Figure 7b, the tint of the background is fixed, and the background itself is almost unrecognizable. The images in Figure 7c show background translations similar to those of RDAGAN. In Figure 7d, flames are generated at the target points, but the localization is poor, which deteriorates object detection performance. Moreover, the images in Figure 7d contain the background degradation. The images in Figure 7e appear to be strongly affected by $L_{BG}^{tr}$, and thus flames are generated in the given areas. However, the shape of the flames indicates that the generator experienced mode collapsed.

Thus, we can confirm that $L_{BG}^{tr}$ is a vital for maintaining the sharpness of the background, $i_{p}$ is crucial for object generation, and $D_{tr}^{local}$ is important for the localization of the generated flame.

#### 5.3.2. Comparison of Generated Objects

**Figure 8:**
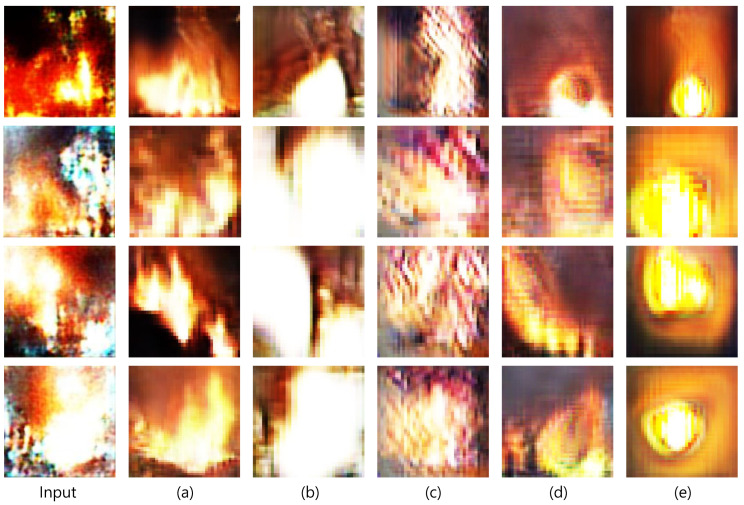
Object generated through RDAGAN and its ablations, (**a**) RDAGAN, (**b**) RDAGAN without $L_{BG}^{tr}$, (**c**) RDAGAN without $i_{p}$ and $L_{Info}^{tr}$, (**d**) RDAGAN without $D_{tr}^{local}$ and $L_{Info}^{tr}$, and (**e**) RDAGAN without $i_{p}$, $D_{tr}^{local}$, and $L_{Info}^{tr}$.

Figure 8 shows the generated objects cropped from Figure 7. The images were sorted in the same order as those in Figure 7. We evaluated the quality of the generated flames and the relations between the inputs and generated flames.

The images in Figure 8a,b are affected by $L_{Info}^{tr}$, whereas those in Figure 8c–e do not. The impact of $L_{Info}^{tr}$ can be determined by evaluating the relationship between the input and output images. Although the input image is not a perfect patch that only requires refinement, RDAGAN generates flame patches while maintaining the characteristics of the input images. The area that appears dark in the generated patch also appears dark in the input image and vice versa. The model that generated the object shown in Figure 8d was provided $i_{p}$ as input; however, the object show less relation with the input because the model did not experience $L_{Info}^{tr}$. The impact of $L_{BG}^{tr}$ can be determined by comparing Figure 8a,b. Owing to $L_{Info}^{tr}$, they exhibit a similar flame pattern, but the lack of $L_{BG}^{tr}$ makes the generated flames in Figure 8b appear unrealistic. The images in Figure 8c demonstrate the importance of $G_{obj}$. The model used to generate images shown in Figure 8c imparted a bright color to the given area, but it failed to synthesize a realistic flame, even though it comprised $D_{tr}^{local}$ that teaches $G_{tr}$ whether the generated object appears like a real flame. In the model used to generate the images shown in Figure 8e, $D_{tr}^{local}$ was removed. The images in Figure 8e show similar shapes and colors. This indicates that mode collapse occurred in the model.

Therefore, we can confirm that $L_{Info}^{tr}$, $L_{BG}^{tr}$, and $D_{tr}^{local}$ play a crucial role in target object generation, and without even one of them, the quality of the generated object is significantly damaged.

## 6. Conclusions

In this paper, we proposed a novel approach, called RDAGAN, to augment image data for object detection models. RDAGAN generates training data for an object detection model using a small dataset. To achieve this, we introduced two subnetworks: an object generation network and an image translation network. The object generation network generates object images to reduce the burden on the image translation network for generating new objects. The image translation network performs image-to-image translation using local and global discriminators. Additionally, we introduced information loss ($L_{Info}^{tr}$) to guide the blending of object patches and clean images, and the background loss ($L_{BG}^{tr}$) to maintain the background information of the clean images.

A quantitative evaluation proved that compared to the original FiSmo dataset, that generated using RDAGAN can enhance the flame detection performance of the YOLOv5 model. In particular, the augmented dataset increased the object localization performance of the YOLOv5 model. Comparative evaluations showed that RDAGAN can not only generate realistic fire images but also confine the area of flame generation, whereas the baseline models cannot. The ablation studies revealed that the absence of one or more components of the RDAGAN can severely damage the model’s generation ability, which indicates the importance of all the components included in the RDAGAN.

In summary, RDAGAN can augment an object detection dataset in a relatively short time and at a low cost without requiring manual collection and labeling of new data to increase the size of the dataset.

## Figures and Tables

**Table 1:** Comparision of the YOLOv5 detection performance.

Dataset	AP@0.5	AP@0.5:0.95	Precision	Recall	F1
FiSmo	0.5082	0.2917	0.5497	**0.5433**	0.5465
**FiSmo + RDAGAN**	**0.5493**	**0.3182**	**0.6922**	0.5173	**0.5921**

## Data Availability

Not applicable.
